# Personal

**Published:** 1918-07

**Authors:** 


### PERSONAL

Announcement of removal, travel, and other matters of interest are requested. Please report errors in the listing of any physician in the State and other directories, that we may co-operate with the State Society in securing a correct list.

Dr. Arthur W. Hurd, for many years Superintendent of the Buffalo State Hospital for the Insane, has resigned and will reside in California.

Dr. Frank W. Ross of Elmira is sick with pneumonia.

Dr. Clarence A. Hill of Buffalo announces the removal of his office to 107 Anderson Pl. He will specialize in anesthesia.

Dr. S. S. Green of Buffalo has returned from Florida and has moved from Niagara St. to 349 Front Ave.

Dr. Lee Masten Francis of Buffalo is to enter the government service as a member of the staff of the hospital for head injuries. The hospital will have from 2,500 to 3,000 beds. The base will be in France.

Dr. Stephen A. Brown of Westfield who was shot in the jaw by Joseph Johnson is now in the Brooks Memorial Hospital and is doing nicely.

Capt. H. B. Huver of Buffalo and Williamsville is attached to a hospital in France, assigned to special service along lines of oral and plastic facial surgery.

Dr. F. B. Willard of Buffalo has moved his office to 317 Richmond Ave.

Dr. Julius Ullman of Buffalo has returned from a visit to Chicago.

